# Repositioning of the Insertable Cardiac Monitor Through the Same Incision to Avoid T-wave Oversensing

**DOI:** 10.7759/cureus.60741

**Published:** 2024-05-21

**Authors:** Shaden Daloub, Alhasan S Alzubi, Khalid Abozguia

**Affiliations:** 1 Internal Medicine, Marshall University Joan C. Edwards School of Medicine, Huntington, USA; 2 Internal Medicine, Mahsa University, Petaling Jaya, MYS; 3 Cardiology, Marshall University Joan C. Edwards School of Medicine, Huntington, USA

**Keywords:** syncopal episodes, icm, t-wave oversensing, insertable cardiac monitor, implantable loop recorders

## Abstract

Insertable cardiac monitor (ICM), used for long-term heart rhythm monitoring, often experiences diagnostic challenges such as T-wave oversensing, leading to false positives. This case report presents a novel approach to rectifying T-wave oversensing in ICM implantations. In this case, we are sharing a 38-year-old female with recurrent syncopal episodes who underwent ICM implantation (LUX-Dx™, ICM-Boston Scientific, Marlborough, United States). Post-implantation, T-wave oversensing was detected. Instead of the usual readjustment or reinsertion, we employed a non-invasive method of repositioning the ICM at a 45-degree angle toward the right side of the heart through the existing incision. This effectively resolved the oversensing issue without complications or the need for a new incision. ICMs are vital in linking symptoms to arrhythmias, especially in cases where standard diagnostic tools fall short. Despite their utility, ICMs are susceptible to T-wave oversensing due to subcutaneous placement. Our case demonstrates a successful alternative approach to address this, enhancing ICM's diagnostic accuracy without invasive procedures. This case highlights the potential of repositioning ICMs as a simple, non-invasive solution to overcome T-wave oversensing issues. It calls for further research and discussion within the medical community to explore its wider applicability, thereby improving ICM efficacy in clinical practice. The patient experienced no complications following the procedure during the three-month visit with appropriate sensing, validating this approach as a feasible option in similar cases.

## Introduction

Insertable cardiac monitors (ICMs) are integral to the continuous monitoring of arrhythmias, representing a paramount technological asset in cardiovascular healthcare. Positioned subcutaneously within the thoracic region, these devices exhibit a commendable capacity for prolonged surveillance, facilitating the detection of cardiac rhythm aberrations over an extended temporal horizon, typically spanning up to three years [1]. Their clinical significance is particularly pronounced in the assessment of recurrent palpitations and unexplained syncope, where ICMs serve as indispensable diagnostic tools in circumstances where transient ambulatory monitoring devices prove inconclusive [2].

Nevertheless, the subcutaneous deployment of ICMs engenders diagnostic intricacies, with oversensing emerging as a salient challenge, predisposing to potential false-positive outcomes [3]. Oversensing not only jeopardizes diagnostic precision but also imposes a dual threat by impinging significantly upon the finite storage capacity of these devices, thereby impeding their overall functional integrity [4]. The gravity of this concern is eloquently underscored by O'Shea et al. in an extensive multicenter cohort study, revealing a disconcerting 59.8% false-positive rate in ICM alerts, with a meager 40.2% representing authentic positives [5]. This dissonance underscores the imperativeness of formulating and implementing strategic modalities for the effective management and mitigation of false-positive alerts in the domain of ICMs.

In response to this exigency, our case report introduces an innovative paradigm directed at ameliorating the challenges posed by oversensing. This pioneering methodology not only proffers a streamlined and minimally invasive alternative to conventional interventions but also underscores a potentially efficacious resolution for addressing oversensing intricacies in the context of ICM implantations.

## Case presentation

We present a case of a 38-year-old female without prior medical history, presented with a history of multiple syncopal episodes over the last six months. She presented with a syncopal episode that lasted around a minute and was self-terminated with no prodromal symptoms. She denied any chest pain, shortness of breath, or palpitation. There are no apparent precipitating or aggravating factors noted. There is no family history of sudden cardiac death, and the patient denies heavy alcohol intake or recreational drug use. The symptoms were not captured by the event monitor over a two-week period due to infrequent episodes. On the exam, the patient was oriented and vitally stable with normal S1 and S2 and no audible murmur. The rest of the exam was within normal limits.

**Figure 1:**
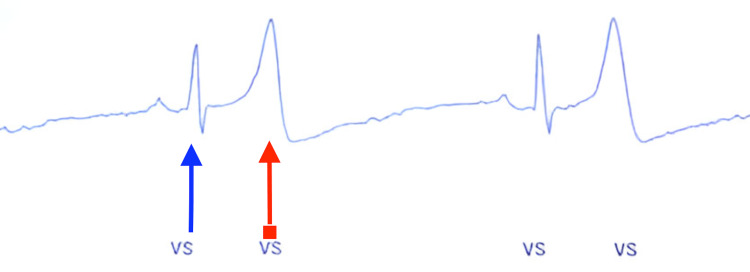
Illustration of T-wave oversensing The blue arrow indicates the appropriate sensing of the ventricle, specifically the QRS complex, while the red arrow points to the inappropriate sensing of the T wave. VS: ventricle sensing

Due to the infrequent symptoms, it was decided to implant an ICM to establish a correlation between symptoms and ECG readings. Throughout the procedure, the heart monitor indicated a normal sinus rhythm. According to the conventional anatomical placement for the ICM, one would expect a clear R wave without any T-wave oversensing. However, the actual outcome in this case was T-wave oversensing as depicted in Figure 1.

**Figure 2:**
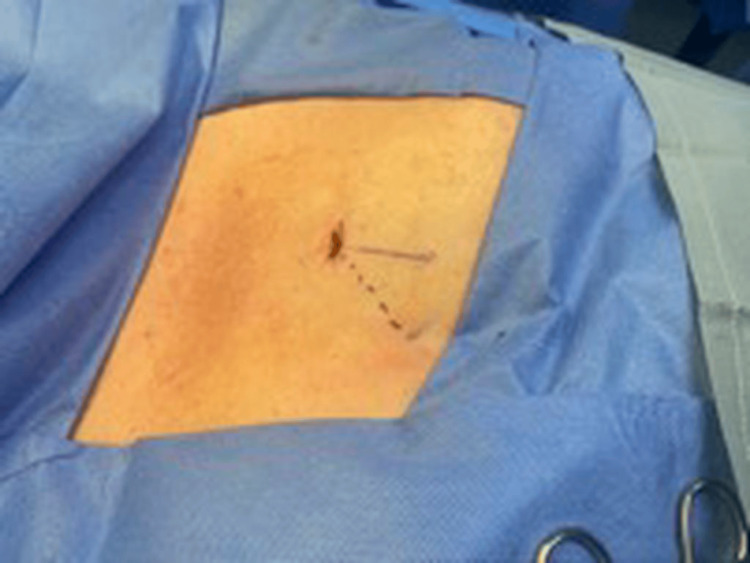
Incision site Solid line: original insertable cardiac monitor (ICM) incision position with T-wave oversensing. Dashed line: repositioning the ICM within the existing incision, adjusting the angle by 45 degrees from the original direction.

Typically, when addressing T-wave oversensing issues in these devices, the common approach involves readjusting the device or, in some cases, removing and reinserting it. However, we propose an alternative, non-invasive method: repositioning the ICM at an angle of 45 degrees to the right side of the heart, away from the left ventricle, through the same incision, thereby eliminating the need for its removal or reinsertion through a new incision (Figure 2).

**Figure 3:**
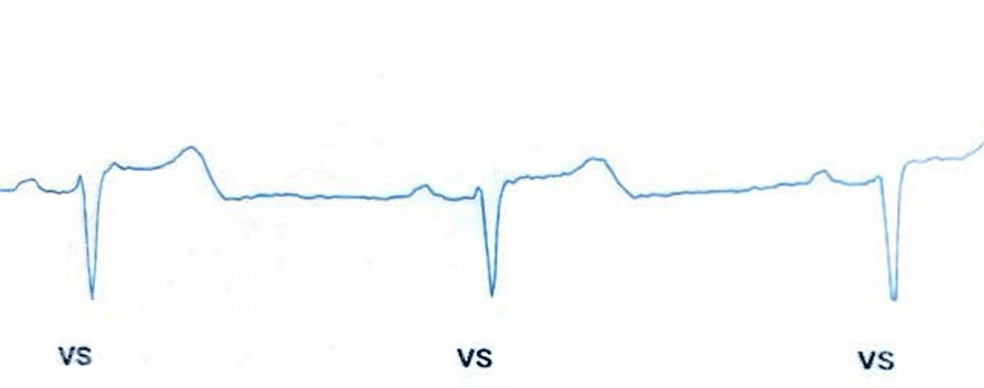
New tracing after the repositioning of the insertable cardiac monitor (ICM), demonstrating appropriate ventricular sensing VS: ventricular sensing

This technique has proven effective in resolving T-wave oversensing (Figure 3).

The patient tolerated the procedure well and was discharged on the same day. After six months of follow-up, monthly remote transmissions did not reveal any issues related to T-wave oversensing.

## Discussion

The primary clinical application of ICMs has proven particularly valuable in patients presenting with recurring syncope, a complex condition characterized by sudden, temporary loss of consciousness. Despite comprehensive initial assessments encompassing clinical history, physical examination, Holter ECG, and echocardiogram, a definitive diagnosis often remains elusive in these cases [6]. To address this diagnostic challenge, ICM devices are meticulously engineered to establish a direct correlation between symptoms and underlying arrhythmias, thereby facilitating the formulation of tailored treatment strategies for affected individuals [7].

However, the clinical utility of ICMs is not without its challenges. Their subcutaneous placement predisposes these devices to potential diagnostic pitfalls, including false positives or missed arrhythmias attributed to interference and electrical noise [3]. Efforts were made to adjust sensitivity; however, they proved unsuccessful in preventing T-wave oversensing. Key among these challenges is the imperative to mitigate instances of "under-sensing" and "over-sensing," both of which can arise from signal variability and may compromise diagnostic accuracy [8].

Subcutaneous placement, though advantageous for patient comfort, can lead to potential false positives or missed arrhythmias due to signal variability and tissue impedance. Additionally, the risk of "under-sensing" or "over-sensing" phenomena necessitates meticulous attention during device implantation and programming to avoid misinterpretation of cardiac rhythms [3]. These challenges underscore the importance of specialized training and expertise in electrophysiology to optimize device placement and maximize diagnostic yield.

The implantation procedure for ICMs typically involves a minor surgical intervention performed on an outpatient basis by a specialized electrophysiologist under local anesthesia. A precise incision, approximately 2 cm from the left side of the sternum and oriented either parallel or at a 45-degree angle to the sternum over the left fourth intercostal space, facilitates the creation of a subcutaneous pocket where the device is securely placed before the incision is closed. This technique usually results in appropriate R-wave sensing [3]. However, in our patient, there was evident T-wave oversensing (Figure 1). In response, we implemented an innovative approach by redirecting the ICM through the same incision away from the apex of the heart. This adjustment successfully avoided T-wave oversensing and restored appropriate R-wave sensing, as depicted in Figure 3. Our case illustrates a simple yet efficient approach that clinicians can employ to mitigate T-wave oversensing.

## Conclusions

This case report demonstrates a novel, less invasive approach to resolving T-wave oversensing in ICMs by repositioning the device at a 45-degree angle toward the right side of the heart through the existing incision. This technique successfully eliminated the need for device removal or reinsertion through alternate incisions, reducing patient discomfort and procedural risks. Avoiding device removal or reinsertion offers substantial potential cost savings and impacts healthcare resource utilization by avoiding the need for patient re-operation, especially if sensitivity adjustment alone fails to resolve T-wave oversensing. Additionally, patients benefit from avoiding multiple incisions, particularly for cosmetic reasons, which is especially significant for younger patients. T-wave oversensing remains a significant challenge for ICM technology, highlighting the continued necessity for innovation to address this issue. Comparative studies comparing sensitivity adjustment and repositioning techniques across diverse patient groups are essential to determining the most effective approach. Feasibility assessments for implementing the repositioning technique in various clinical settings are crucial for identifying potential barriers to adoption. Additionally, developing specific algorithms tailored to eliminate T-wave oversensing could enhance the accuracy and reliability of ICMs in cardiac monitoring. These efforts will advance our understanding of effective management strategies for T-wave oversensing, optimizing the use of ICM devices, and improving patient care and diagnostic accuracy in cardiac monitoring. T-wave oversensing in intracardiac monitoring can result in misdiagnosis of cardiac arrhythmias and unwarranted interventions, potentially leading to patient distress and heightened healthcare utilization. The case underlines the increasing concerns regarding false-positive alerts in ICMs, a significant issue in their clinical utility. It suggests a promising solution for similar clinical scenarios and emphasizes the importance of further research and dialogue within the medical community to explore non-invasive methods that enhance the efficacy and safety of cardiac monitoring devices. This contribution is a step toward refining ICM technology for better patient outcomes, inspiring more innovation in the field.
